# Strychnine poisoning due to traditional Chinese medicine: a case series

**DOI:** 10.12688/f1000research.73072.2

**Published:** 2021-12-17

**Authors:** Hok-Fung Tong, Candace Yim Chan, Sau-Wah Ng, Tony Wing-Lai Mak

**Affiliations:** 1 Hospital Authority Toxicology Reference Laboratory, Princess Margaret Hospital, Hospital Authority, Hong Kong

**Keywords:** Strychnine poisoning, herbal medicine poisoning, Strychni semen, Maqianzi

## Abstract

**Background:** Strychnine poisoning is rare but possibly fatal. The most reported sources of strychnine poisoning include rodenticides and adulterated street heroin. Here we report a case series of an unusual cause of strychnine poisoning – *Strychni* *semen*, a herb known as “maqianzi” in traditional Chinese medicine (TCM).

**Methods:** All cases of strychnine poisoning confirmed by the Hospital Authority Toxicology Reference Laboratory (HATRL, the highest-level clinical toxicology laboratory in Hong Kong) between May 2005 and May 2018 were reviewed.

**Results: **Twelve cases of strychnine poisoning were recorded, and *Strychni semen *was the exclusive source. Ten (83%) patients presented with muscle spasms, and four (33%) developed typical conscious convulsions. The poisoning was severe in two (17%) patients, moderate in three (25%) and mild in eight (58%). No case fatality was recorded. Three (25%) patients were TCM practitioners and two (17%) were laymen who bought the herb themselves without a proper prescription.

**Conclusion:** The practice of TCM is becoming popular in different parts of the world amid the COVID-19 pandemic. The spectrum of clinical features of strychnine poisoning secondary to *Strychni semen* are similar to those arising from different origins. Eliciting a history of TCM use, apart from exposure to rodenticides and drugs of abuse, may allow timely diagnosis in patients with compatible clinical features. Enhancement of TCM safety could minimize the hazard.

## Background

Around a century ago, the wide availability of strychnine as an over-the-counter remedy for a variety of ailments caused a significant number of deaths, especially in children ^1^. As such, strychnine has been withdrawn from the food and medicine markets in many countries, and poisoning is now rare. Nowadays, the most reported sources of poisoning include rodenticides ^2– 4^ and adulterated street heroin ^5– 7^.

*Strychni semen*, a herb also known as “maqianzi” in traditional Chinese medicine (TCM), also contains strychnine together with its less toxic analog, brucine. The herb is used for its analgesic, anti-diarrheal, antioxidant, anti-inflammatory, anti-microbial and anti-neoplastic properties ^8, 9^. However, it has an apparently narrow therapeutic index – the recommended dosage is 0.3–0.6 g daily according to the Chinese Pharmacopoeia 2015 ^10^.

Strychnine is rapidly absorbed from the gastrointestinal tract and toxicity may occur as soon as 15 minutes after ingestion. By inhibition of glycine receptors at the spinal cord, strychnine overdose causes stiffness and intermittent spasms. Each episode of spasm lasts for around half to two minutes and is easily triggered by emotional distress or even trivial sensory stimuli ^11^. Severe spasms may mimic epileptic convulsions, but there is preserved awareness without post-ictal drowsiness. This typical feature of strychnine poisoning is known as conscious convulsions/spinal seizures ^12^ and may mimic tetanus, though the latter can be differentiated by a positive history of injury, and a more gradual and protracted course of illness ^13^. Severe convulsions may cause rhabdomyolysis and can be rapidly fatal due to respiratory failure ^14^. Milder symptoms of strychnine poisoning include agitation, palpitations, hyperventilation, dizziness, hyperacusis and paresthesia ^15^.

Apart from epilepsy and tetanus, acute dystonia and hypocalcemia are the other differential diagnoses of strychnine poisoning. In acute dystonia, muscle contractions are more static. Hypocalcemia also results in numbness and muscle spasms but can be easily differentiated by blood tests ^16^.

Management of strychnine poisoning is supportive. Activated charcoal may be given if patients present early. Benzodiazepines are used to control muscle spasms, and a dark and quiet environment also helps. Airway intervention and mechanical ventilation can be lifesaving, though succinylcholine must be avoided as it exacerbates muscle spasms ^16– 18^.

Here we report a case series of an unusual cause of strychnine poisoning – *Strychni semen*.

## Methods

All cases of strychnine poisoning, which were analytically confirmed by the Hospital Authority Toxicology Reference Laboratory (HATRL), between May 2005 and May 2018 were reviewed. HATRL is the highest-level clinical toxicology laboratory serving all public hospitals in Hong Kong, and it is the only laboratory which provides analytical confirmation of strychnine poisoning in the territory. Clinical records were reviewed to collect the patient demographics, details of exposure, clinical features, pertinent laboratory findings, treatment provided and clinical outcomes.

Diagnosis of strychnine poisoning was confirmed by detection of strychnine in urine, and *Strychni semen* exposure was inferred by the concurrent detection of brucine. Detection of strychnine and brucine was performed by using high-performance liquid chromatography with diode array detection (HPLC-DAD; Agilent 1100 and 1200 LC Systems with DAD) and liquid chromatography-tandem mass spectrometry (LC-MS/MS; Agilent 1100 LC System with Applied Biosystems 4000 QTrap triple-quadruple mass spectrometer) ^19^. Wherever available, herbal prescriptions were transcribed and studied, unused herbs were morphologically identified, and analysis of strychnine and brucine was performed on suspicious herbal products.

## Results

 There were twelve cases of strychnine poisoning confirmed during the study period, and all of them were due to *Strychni semen* ( Table 1). Two severe cases are further described to highlight the salient features.

**Table 1.** Summary of patient demographics and clinical features.

Case	Year	Sex	Age (y)	Latent period (hrs)	Symptom duration (hrs)	Stiffness	Spasm	Convulsion	Other manifestations
**1** ^c^	2005	M	58	0.5	4.5	-	-	-	Agitation, palpitations, dizziness, paresthesia
**2** ^b^	2005	M	52	0.6	1.0	+	+	-	Not reported
**3**	2005	F	36	2.0	4.0	+	+	-	Not reported
**4** ^b^	2006	M	32	0.5	0.5	+	+	-	Palpitations, dizziness
**5** ^*, b^	2006	M	55	0.5	6.0	+	+	+	Dizziness, hyperventilation
**6** ^a^	2006	F	8	0.5	0.5	+	-	-	Paresthesia
				0.2	1.3	+	+	-	Dizziness, paresthesia
**7** ^*^	2007	M	45	1.0	7.0	+	+	+	Dyspnea, paresthesia, dizziness, hyperacusis
**8** ^a, c^	2007	F	54	2.0	N/A	-	+	-	Dizziness
				2.0	6.0	+	+	+	Vomiting, dizziness
**9**	2007	F	36	0.3	3.0	+	+	+	Palpitations
**10** ^a^	2008	M	49	N/A	3.0	-	-	-	Chest pain, dyspnea
				1.0	3.0	-	-	-	Chest pain, dyspnea, respiratory acidosis
**11**	2012	F	55	0.5	6.0	-	+	-	Paresthesia
**12** ^a^	2018	F	48	0.5	3.0	-	+	-	Palpitations, dyspnea
				2.0	3.0	+	+	-	Palpitations, dyspnea

*: Severe poisoning (see text for description) ^a^: Recurrent episodes of poisoning ^b^: This patient was a TCM practitioner ^c^: This patient was a layman who bought the herb from a TCM pharmacy ^+^: Present ^-^: Absent

A 55-year-old male (case 5 in Table 1), a TCM practitioner, was woken up by severe wrist pain at night and inadvertently took 19 grams (31 times the recommended therapeutic dosage) of *Strychni semen* instead of another herb. He developed dizziness, hyperventilation, generalized muscle spasms and conscious convulsions 30 minutes later. He attended the accident & emergency department and was hospitalized. Activated charcoal and intravenous diazepam (3 mg) were given, and the symptoms subsided six hours later. However, there was rhabdomyolysis (plasma creatine kinase level 5085 U/L; reference interval 45–235 U/L) and he required prolonged aggressive rehydration. *Strychni semen* exposure was confirmed by detection of both strychnine and brucine in urine. He eventually recovered and was discharged after one week of admission.

A 45-year-old man (case 7 in Table 1) consulted a bone setter for gouty arthritis and was dispensed some herbal powder with undisclosed ingredients. One hour after taking the herbal powder, he developed chest discomfort, dyspnea, hyperacusis, generalized muscle spasms, trismus, opisthotonus and conscious convulsions. He was hospitalized and repeated dosing of intravenous diazepam (10 mg in total) was required to control the convulsions. The symptoms lasted for seven hours, but he soon requested early discharge despite medical advice. *Strychni semen* exposure was subsequently analytically confirmed.

The demographics and clinical features of all 12 patients are summarized in Table 1 in chronological order of presentation. Numbers of male and female patients were equal. The median age was 48.5 years (range 8 – 58 years). *Strychni semen* exposure was the exclusive source of strychnine poisoning, and the herb was most commonly used to relieve chronic pain. Four (33%) patients had recurrent episodes of poisoning before the definite diagnosis was made. Ten (83%) patients presented with muscle spasms, and four (33%) patients developed typical conscious convulsions. One patient (case 10) had transient respiratory acidosis, which resolved spontaneously. Median latent period before symptom onset was 0.5 hours (range 0.2 – 2 hours), and the median symptom duration was 3.5 hours (range 0.5 – 7 hours). Based on a previously published poisoning severity score system ^20^, the poisoning was severe in two (17%) patients, moderate in three (25%) and mild in seven (58%). No case fatality was recorded, and all patients recovered eventually.

Intriguingly, three (25%) patients were professional TCM practitioners (cases 2, 4, 5) themselves. On the other hand, two (17%) were laymen who bought *Strychni semen* without a proper prescription. One was a housewife (case 8) who bought the herb at a Chinese medicine pharmacy and took a markedly excessive dose (15 times the recommended dosage) of *Strychni semen* based on a misprinted soup recipe in a book borrowed from a public library ^21^.

## Discussion

Our results showed that *Strychni semen* was the exclusive source of clinically and analytically confirmed strychnine poisoning in Hong Kong during the 14-year study period. In addition, *Strychni semen* poisoning shared a similar spectrum of clinical features with strychnine poisoning due to other causes ^1, 11, 12, 16, 17, 22^. This peculiarly predominant source of strychnine poisoning in Hong Kong is likely contributed to by the fact that most Hong Kong residents are ethnic Chinese.

Nevertheless, with globalization, immigration and the rising popularity of TCM around the world ^23^, especially amid the COVID-19 pandemic ^24^, eliciting a history of herbal medicine use, in addition to exposure to rodenticides and drugs of abuse, may be helpful in making the clinical diagnosis of strychnine poisoning. Identifying the source of strychnine poisoning is also important for public health measures.

Unfortunately, obtaining a definite history of *Strychni semen* use can be challenging if the herbal prescription is not disclosed by the TCM practitioner. In our case series, five (42%) patients did not possess such information, which made the diagnosis difficult. We noted that TCM clinics associated with the Hospital Authority and the universities in Hong Kong provide computer-generated herbal prescriptions to their patients. However, this good practice is neither universally exercised nor required legally.

Intriguingly, three patients of our case series were TCM practitioners themselves, who were the learned professionals of this dangerous herb. This illustrates the fact that *Strychni semen* is a dangerous herb and systematic measures should be introduced to minimize such poisoning events.

Enhancement in TCM dispensing could be an important measure. Among our case series, the TCM dispensers did not prevent the inappropriate dosages of *Strychni semen* from being prescribed by the TCM practitioners. Shockingly, *Strychni semen* was dispensed to two patients (case 1 & 8) who did not bear a registered TCM practitioner’s prescription. These suboptimal practices should certainly be noted and enhanced by the profession. To ensure patient safety, both good prescription and dispensing practices are essential. In the long run, all TCM practitioners and dispensers around the world should be adequately trained, certified and licensed.

## Conclusion

The practice of TCM is becoming popular in different parts of the world amid the COVID-19 pandemic. This study serves to remind the profession that strychnine poisoning can result from TCM use. The spectrum of clinical features of strychnine poisoning secondary to *Strychni semen* (maqianzi) are similar to those that arise from different origins. Eliciting a history of TCM use, apart from exposure to rodenticides and drugs of abuse, may allow timely diagnosis in patients with compatible clinical features. Enhancement of TCM safety could minimize the hazard.

## Consent

We respected all patients’ rights to privacy and protected their identity. The study was approved by the Kowloon West Cluster Research Ethics Committee of the Hospital Authority, Hong Kong (approval number KW/EX-19-002), who waived the need for informed consent as the presented data have been anonymized and de-identified.
