# Supplementary material for: Strychnine poisoning due to traditional Chinese medicine: a case series
Source: F1000Res. 2021 Dec 17;10:924. Originally published 2021 Sep 15. [Version 2] doi: 10.12688/f1000research.73072.2 (PMC8817065; doi:10.12688/f1000research.73072.2)
Supplement: Peer review history

# Peer review: Reviewer response for version 2

**DOI:** 10.5256/f1000research.80788.r115743

**Published:** 2022-02-04

**Authors:** Camille Paradis

**Affiliations:** 1 Centre Hospitalier Universitaire de Bordeaux, Bordeaux, France

Thank you very much for your responses and changes to your manuscript, I fully agree with them.

Are enough details provided of any physical examination and diagnostic tests, treatment given and outcomes?

Partly

Is the background of the cases’ history and progression described in sufficient detail?

Partly

Is sufficient discussion included of the importance of the findings and their relevance to future understanding of disease processes, diagnosis or treatment?

Partly

Is the conclusion balanced and justified on the basis of the findings?

Yes

Reviewer Expertise:

NA

I confirm that I have read this submission and believe that I have an appropriate level of expertise to confirm that it is of an acceptable scientific standard.

# Peer review: Reviewer response for version 1

**DOI:** 10.5256/f1000research.76695.r101016

**Published:** 2021-12-06

**Authors:** Camille Paradis

**Affiliations:** 1 Centre Hospitalier Universitaire de Bordeaux, Bordeaux, France

This is a retrospective case series. This work is very interesting since it presents a circumstance of exposure to strychnine by therapeutic error, which is unusual compared to the numerous cases of intoxication in the context of suicidal behavior or other involuntary circumstances. This has implications for the management of intoxicated patients because this circumstance does not raise suspicion of this type of xenobiotic.

 For the context, I find that the reason for the use of strychnine as a heroin adulterant is not easy to understand. If it is difficult to explain it quickly, perhaps it would be better to just mention this use, without detailing its origin. The bibliographic reference can remain the same. Secondly, I did not really understand why the term "weakness" was used for the description of strychnine poisoning. Classically, patients present with a state of hyperexcitability with significant rigidity, perhaps you meant to refer to the symptom "coma" by this term? If you change it, consider doing so in Table 1 as well.

 Regarding the data shared on your case series, do you have any notion of the amounts of strychnine and brucine found in the urine of your patients? If you have them, it would be a pity not to share them, especially to compare them with the rest of the literature. In addition, it might be interesting to provide the dates of each case of intoxication, perhaps in your table. Actually, you indicate that the use of TCM is increasing, it might be interesting to see how the number of intoxications evolve over the duration of your study.

 For the discussion, I really have trouble seeing how you conclude that *Strychni semen* poisonings are the only circumstances of strychnine poisonings in your study location. It is possible that you have other arguments for stating this, but they need to be included in your paper. In particular, you should explicitly state that no center other than HATRL can analytically confirm strychnine poisoning in your study location. In a recently published study by *Paradis et al.,* ^1^ it is shown that cases of strychnine poisoning still occur through old rodenticide products. Then I find the sentence that starts with "We believe that empowering patients..." perhaps a bit long and I find it difficult to easily understand its purpose.

 Finally, two formal remarks. On the bibliography, you specify "Clin Toxicol (Phila)" (2) and "Clin Toxicol" alone (13), check that there is not a clarification to be made on which one is alone. For the expression of all your values as a percentage, it seems to me that for a number of patients <100 you should express your values as integers and not as decimal numbers.

 For the conclusion, I find it perfectly appropriate with a clear purpose, its opening is particularly valid.

Are enough details provided of any physical examination and diagnostic tests, treatment given and outcomes?

Partly

Is the background of the cases’ history and progression described in sufficient detail?

Partly

Is sufficient discussion included of the importance of the findings and their relevance to future understanding of disease processes, diagnosis or treatment?

Partly

Is the conclusion balanced and justified on the basis of the findings?

Yes

Reviewer Expertise:

Pharmacy, Clinical Toxicology

I confirm that I have read this submission and believe that I have an appropriate level of expertise to confirm that it is of an acceptable scientific standard, however I have significant reservations, as outlined above.

## References

[1] : Strychnine, old still actual poison: description of poisoning cases reported to French Poison Control Centers over the past thirteen years. *Toxin Reviews* .2021; 10.1080/15569543.2021.1983843 1-7 10.1080/15569543.2021.1983843

# Response

**Published:** 2021-12-09

**Authors:** Hok Fung Tong

Thank you very much for your comments and suggestions on improving the quality of the manuscript. Please kindly find below our responses.

 1) For context, I find that the reason for the use of strychnine as a heroin adulterant is not easy to understand. If it is difficult to explain it quickly, perhaps it would be better to just mention this use, without detailing its origin. The bibliographic reference can remain the same.

 - The reason has been removed.

 2) I did not really understand why the term "weakness" was used for the description of strychnine poisoning. Classically, patients present with a state of hyperexcitability with significant rigidity, perhaps you meant to refer to the symptom "coma" by this term? If you change it, consider doing so in Table 1 as well.

 - We replaced the term “weakness” with “stiffness” instead as this should be the more appropriate description, although some patients would describe such symptom as “weak”.

 3) Regarding the data shared on your case series, do you have any notion of the amounts of strychnine and brucine found in the urine of your patients? If you have them, it would be a pity not to share them, especially to compare them with the rest of the literature.

 - Our laboratory did not provide quantitative analyses of strychnine and brucine. Therefore, such information is not available.

 4) In addition, it might be interesting to provide the dates of each case of intoxication, perhaps in your table. Actually, you indicate that the use of TCM is increasing, it might be interesting to see how the number of intoxications evolve over the duration of your study.

 - We have added the years of clinical presentation in Table 1.

 5) For the discussion, I really have trouble seeing how you conclude that Strychni semen poisonings are the only circumstances of strychnine poisonings in your study location. It is possible that you have other arguments for stating this, but they need to be included in your paper. In particular, you should explicitly state that no center other than HATRL can analytically confirm strychnine poisoning in your study location.

 - Being the highest level clinical toxicology laboratory in Hong Kong, HATRL is the only laboratory which provides analytical confirmation for strychnine poisoning locally. For all analytically confirmed strychnine poisoning cases that we have encountered during the study period, they were all due to Strychni semen. Such information is now stated in the methods section.

 6) In a recently published study by Paradis et al., it is shown that cases of strychnine poisoning still occur through old rodenticide products.

 - We agree that strychnine poisoning can still occur through rodenticides, and have added the citation of this study in the introduction section.

 7) Then I find the sentence that starts with "We believe that empowering patients..." perhaps a bit long and I find it difficult to easily understand its purpose.

 - We have removed the sentence.

 8) Finally, two formal remarks. On the bibliography, you specify "Clin Toxicol (Phila)" (2) and "Clin Toxicol" alone (13), check that there is not a clarification to be made on which one is alone. For the expression of all your values as a percentage, it seems to me that for a number of patients <100 you should express your values as integers and not as decimal numbers.

 - We have unified the references as “Clin Toxicol”, and have modified the percentages accordingly.

# Peer review: Reviewer response for version 1

**DOI:** 10.5256/f1000research.76695.r94448

**Published:** 2021-09-29

**Authors:** Jou-Fang Deng

**Affiliations:** 1 Division of Clinical Toxicology and Occupational Medicine, Department of Medicine, Taipei Veterans General Hospital, Taipei, Taiwan

I am a medical toxicologist having been in practice since 1982. Many years ago, I used to treat severe convulsion cases related to events of drug abuse, which ended up with a diagnosis of strychnine poisoning confirmed by a clinical toxicology laboratory. However, strychnine poisoning related to the use of traditional medicine is not what I am familiar with, and as it is described in the article, it indeed may potentially occur in our society, in which the practice of traditional medicine is not well regulated.

 Strychnine poisoning could be a life-threatening condition, and without appropriate treatment and timely ventilation support, life can disappear quickly. This article has brought up some important points, relevant to both the societies of traditional medicine and western medicine. Since strychnine itself does possess prominent toxicity, to prevent any unnecessary and avoidable poisoning, I would like to propose that the dispensing of strychnine contented herbs should not continue, given its toxic potential.

 This article describes a retrospective study of a collection of unusual cases of strychnine poisoning which need to be remembered in general practice as well as emergency settings. This is a very interesting clinical report and is valuable for the clinical doctors. Since it is a well written article, and clearly demonstrates the importance of the history of herb intake in our daily practice, I would recommend that there is no need for any revision.

Are enough details provided of any physical examination and diagnostic tests, treatment given and outcomes?

Yes

Is the background of the cases’ history and progression described in sufficient detail?

Yes

Is sufficient discussion included of the importance of the findings and their relevance to future understanding of disease processes, diagnosis or treatment?

Yes

Is the conclusion balanced and justified on the basis of the findings?

Yes

Reviewer Expertise:

Medical toxicologist

I confirm that I have read this submission and believe that I have an appropriate level of expertise to confirm that it is of an acceptable scientific standard.
